# Polyurethane Adhesives with Chemically Debondable Properties via Diels–Alder Bonds

**DOI:** 10.3390/polym16010021

**Published:** 2023-12-20

**Authors:** María Pilar Carbonell-Blasco, María Alejandra Moyano, Carlota Hernández-Fernández, Francisco J. Sierra-Molero, Isidro M. Pastor, Diego A. Alonso, Francisca Arán-Aís, Elena Orgilés-Calpena

**Affiliations:** 1 Footwear Technology Centre, Campo Alto Campo, Elda, 03600 Alicante, Spain; amoyano@inescop.es (M.A.M.); chernandez@inescop.es (C.H.-F.); aran@inescop.es (F.A.-A.); eorgiles@inescop.es (E.O.-C.); 2 Department of Organic Chemistry, Institute of Organic Synthesis (ISO), Faculty of Sciences, University of Alicante, P.O. Box 99, 03080 Alicante, Spain; francisco.sierra@ua.es (F.J.S.-M.); ipastor@ua.es (I.M.P.); diego.alonso@ua.es (D.A.A.)

**Keywords:** adaptable covalent networks, polyurethane, adhesives, Diels–Alder reaction, sustainability

## Abstract

Covalent adaptable networks (CANs) represent a pioneering advance in polymer science, offering unprecedented versatility in materials design. Unlike conventional adhesives with irreversible bonds, CAN-based polyurethane adhesives have the unique ability to undergo chemical restructuring through reversible bonds. One of the strategies for incorporating these types of reactions in polyurethanes is by functionalisation with Diels–Alder (DA) adducts. By taking advantage of the reversible nature of the DA chemistry, the adhesive undergoes controlled crosslinking and decrosslinking processes, allowing for precise modulation of bond strength. This adaptability is critical in applications requiring reworkability or recyclability, as it allows for easy disassembly and reassembly of bonded components without compromising the integrity of the material. This study focuses on the sustainable synthesis and characterisation of a solvent-based polyurethane adhesive, obtained by functionalising a polyurethane prepolymer with DA diene and dienophiles. The characterisation of the adhesives was carried out using different experimental techniques: nuclear magnetic resonance spectroscopy (NMR), Brookfield viscosity, differential scanning calorimetry (DSC), thermogravimetric analysis (TGA) and T-peel strength testing of leather/adhesive/rubber joints to determine the adhesive properties, both before and after the application of external stimuli. The conversion of both the DA and retro-Diels–Alder (r-DA) reactions was confirmed by ^1^H-NMR. The adhesive properties were not altered by the functionalisation of the adhesive prepolymer, showing similar thermal resistance and good rheological and adhesive properties, even exceeding the most demanding technical requirements for upper-to-sole joints in footwear. After the application of an external thermal stimuli, the bonded materials separated without difficulty and without damage, thus facilitating their separation, recovery and recycling.

## 1. Introduction

Nowadays, there is a shortage of technologies designed to efficiently disassemble a shoe for the subsequent recycling of its various components once it has reached the end of its useful life. Some approaches focus on mechanical shredding and subsequent separation into fractions according to density, but this can result in polymer mixtures, occasionally with a certain degree of degradation. Consequently, current efforts in the industry aim at providing adhesive bonds with the ability to be reversed on demand. To achieve this, the typical methods are the addition of dismountable agents (i.e., nanoparticles, thermally expandable particles, additives, etc.) and the chemical modification of the polymer chain by introducing reversible bonds [1,2].

The creation of reversible adhesives, which is achieved by modification of the polymer chain with the introduction of reversible bonds, is based on the ability to untwist the polymer chain when exposed to a specific external stimulus (i.e., heat, pressure, UV light, radio frequencies, etc.). This allows for a temporary separation of the adhesive bond [3,4,5,6,7].

Specifically, polyurethane adhesives are the most widely used in the footwear sector due to their great versatility for joining materials of different natures. These adhesives are chemically, thermally and mechanically stable. Due to their covalent bonds, they create crosslinking structures that provide strong bonds between the adhered materials. However, these networks do not allow the bonded materials to be separated, reprocessed, remodelled or recycled after their useful life [8].

One potential strategy for recycling materials that are bonded with polyurethane adhesives is the incorporation of dynamic covalent bonds into the adhesives, which are known as covalent adaptive networks (CANs) [9,10]. These bonds are reversible under certain conditions, which are achieved by stimulation (temperature, humidity, mechanical stress, light or pH) [11,12,13]. The incorporation of these dynamic links can be performed in different ways, including transesterification, DA/r-DA chemistry, imide nods, disulfide metathesis, dynamic B-O bonds, hemiaminals/hexahydrotriazines and acetal linkages [14,15,16]. CANs exhibit a wide variety of physical characteristics, including adaptability, self-healing properties, shape and stimulus memory, responsiveness to stimuli and enhanced recyclability. The inherent nature of the dynamic bonds of CANs determines their physical characteristics and operating conditions such as temperature, pH and humidity [17].

The Diels–Alder reaction (DA) is noteworthy for being an extensively utilised methodology in the chemical industry. Nowadays, its incorporation in the polymer industry is starting to be explored due to its responsiveness to thermal stimuli. This unique feature allows for its effective application across a broad range of industrial uses. In the case of polyurethane chemistry, the electron-rich furan/electron-poor maleimide stands out as the prevalent diene/dienophile pair in DA reactions due to its wide availability and to the fact that it is the origin of furan derivatives from renewable sources. This cycloaddition reaction occurs across a wide temperature range, allowing the application of this reversible transformation in the preparation of dynamic materials. At approximately 60 °C, the formation of long-lasting adducts in DA additions takes place, while the reverse reaction (i.e., retro-Diels–Alder (r-DA)) begins from 110 °C. These properties allow DA reactions to play an increasingly relevant role in promoting sustainable practices within the realms of green chemistry and materials science [18]. In particular, the thermal reversibility of the DA reaction and the mild conditions usually involved in the r-DA reaction render this reversible transformation particularly suitable for creating polymeric materials, enabling the separation of on-demand bonded components by heat stimulation [19,20].

In this regard, our study involves the sustainable development of stimuli-responsive polyurethane (PU) adhesives by incorporating thermo-reversible Diels–Alder bonds. The incorporation of DA bonds into the adhesive allowed us to adjust the adhesive characteristics by breaking the DA bonds through a r-DA reaction through application of temperature.

Recently, there has been a growing interest in the integration of DA reactions in polyurethane matrices [21,22]. This trend is mainly focused on the development of materials that are capable of performing self-healing processes [23,24,25]. However, only a few studies have explored different applications, such as microencapsulation [26,27] or recycling [28,29]. For instance, Heo et al. developed two new thermo-responsive self-repairing polyurethanes based on the DA reaction between furan and maleimide molecules, which use the shape memory effect to bring the crack faces into intimate contact, thus enabling their repair. Both polyurethanes presented a stable polymer structure and mechanical properties that were comparable to those of commercial epoxies. The results of such research showed that the shape memory effect can replace external forces to close two crack surfaces and that the DA reaction could be used repeatedly to heal the cracks [30].

Moreover, Chen et al. synthesised a triol-functional crosslinker that combined the thermo-reversible properties of DA adducts as an ideal substitute for a traditional crosslinker to prepare thermally recyclable crosslinked polyurethanes with excellent mechanical and recyclable properties [31]. In addition, Wang et al. introduced dynamic sodium carbamate bonds into a crosslinked polyurethane network to obtain a new type of hot-melt polyurethane adhesive. These adhesives exhibited fast curing, excellent initial and final adhesive strength, and a high tolerance to product solvents. They also showed the ability to adhere and self-heal, given the dynamic nature of their polymeric network [32,33].

Against this background, our work aims at developing new reversible polyurethane adhesives by creating and breaking tailor-made bonds in order to reduce the impact caused by the end-of-life of adhesives and final footwear products. To achieve this aim, our focus was on obtaining polyurethane adhesives functionalised with Diels–Alder adducts in the prepolymer. For this purpose, four adhesives were developed using solvent-free sustainable conditions, one of which was an unmodified polyurethane adhesive, and the other three incorporated DA adducts in the prepolymer. These four PU adhesives were fully characterised and studied before and after the application of an external thermal stimulus to determine the adhesives’ behaviour with the DA and r-DA reactions.

## 2. Materials and Methods

### 2.1. Materials

Polyurethanes were synthetised using 1,4-butanediol polyadipate (Hoopol F-580 Synthesia Technology, Barcelona, Spain) (Mw= 3000 g/mol, IOH = 37–40 mg KOH/g) as polyol and 4,4-diphenylmethane diisocyanate (MDI) (Sigma Aldrich, Barcelona, Spain) as isocyanate. Furfurylamine (Sigma Aldrich, St. Louis, MO, USA) and 1,1′-(methylenedi-4,1-phenylene) bismaleimide (BMI) (BLDpharm, Kaiserslautern, Germany) were utilised for the functionalisation of the adhesive. 1,4-butanediol (99% purity, Thermo Scientific Chemicals, Madrid, Spain) was used as chain extender and Desmodur RFE (Bayer, Leverkusen, Germany) as crosslinker.

### 2.2. General Procedure for the Functionalisation of Polyurethane by DA/r-DA Reaction

The functionalisation of polyurethane adhesives was carried out under neat conditions (i.e., in the absence of added solvent) using the prepolymer method with furan-type dienes for the study of the thermal Diels–Alder (DA)/retro-Diels–Alder (r-DA) reaction with bismaleimides [34,35]. The conversion of each reaction step was controlled via ^1^H-NMR analysis. Thus, the PU prepolymer was obtained under an Ar atmosphere in a 250 mL four-hole glass reaction vessel, equipped with a stirrer with a crossed stainless steel rod, by reaction of 1,4-butanediol polyadipate (100 g, 33.3 mmol), which had been previously dried under vacuum at 45 °C for 16 h (to remove moisture) with MDI (11.37 g, 46.7 mmol, for a NCO/OH ratio of 1.4) under neat conditions at 60 °C for 1 h. Once the prepolymer was formed, a sample of approximately 1 g was extracted to determine the percentage of free isocyanate through back-titration with dibutylamine. The prepared prepolymer was then functionalised in situ with furfurylamine (2.4 mL, 26.70 mmol), which was slowly added to the prepolymer at 60 °C. After 1 h, the functionalisation was completed, and BMI (4.7850 g, 13.35 mmol) was subsequently added to the polymer to carry out the DA cycloaddition process at 60 °C for 16 h. The resulting polymer was cut into small pieces to facilitate its subsequent dissolution in an appropriate organic solvent (18 ± 2 wt% in methylethylketone, Quimidroga, Barcelona, Spain), thus obtaining the polyurethane adhesive. Finally, the thermal reversibility of the DA process (r-DA) was studied under two different reaction conditions. First, the r-DA reaction was carried out in standard conditions by simply raising the temperature of the interior of the reaction vessel to 110 and 130 °C. After verifying that the r-DA reaction had occurred satisfactorily, the effect of performing the r-DA in a solvent medium was evaluated. For this end, upon completion of the DA reaction, small samples (approximately 0.5 g) of the polymer were taken and dissolved in methyl ethyl ketone (MEK) in a polymer/MEK ratio of 18/82 (wt%). The solution obtained was placed as a thin film over aluminium foil, which was heated on a hot plate for 24 h. After this time, the polymer was collected from the aluminium foil and analysed by ^1^H-NMR.

### 2.3. Characterisation Techniques

Nuclear magnetic resonance spectroscopy (NMR): NMR spectra were recorded for ^1^H-NMR at 300 or 400 MHz on a Bruker AC-300 or AC-400 instrument from the Technical Services (SSTTI) of the University of Alicante (UA), using CDCl_3_ as solvent and TMS (0.0003%) as internal standard, unless otherwise indicated. Chemical shifts (*δ*) were expressed in units of parts per million, relative to TMS, and coupling constant (*J*) in Hertz (Hz). The multiplicity of signals was indicated as follows: s (singlet), d (doublet), t (triplet), dd (doublet of doublets), m (multiplet).

Brookfield viscosity: The viscosity of the dispersions was determined using a Brookfield RVT DV Next viscometer (Brookfield, Middleboro, MA, USA). For all measurements, a number 5 rod was used, and a sweep of different rotational speeds was performed at room temperature.

Solids content (UNE-EN 827:2006): The solids content was determined by evaporating the volatile components at 100 °C to constant mass [36].

Differential scanning calorimetry (DSC) was employed to determine the thermal properties of polyurethane adhesives with a DSC3 + STAR^e^ Systems calorimeter (Mettler-Toledo AG, Schwerzenbach, Switzerland). The experiments were conducted in an inert nitrogen atmosphere (flow rate = 30 mL min^−1^) at a heating rate of 10 °C/min. The optimal conditions for DSC experiments had been optimised by the authors in a previous work [37].

Thermogravimetric analysis (TGA) was employed to study the thermal stability of polyurethane hot-melt adhesives with a TGA 2 STAR^e^ System thermal balance, equipped with STAR^e^ software 16.30 (Mettler-Toledo AG, Schwerzenbach, Switzerland). The sample was heated from 30 °C to 600 °C in 10 °C/min in an inert nitrogen atmosphere (flow rate = 30 mL min^−1^) [38].

T-peel strength test: The adhesive properties of polyurethane adhesives were determined on split leather/SBR rubber joints, which are common upper and sole materials in the footwear industry, following the procedure described in standard EN 1392:2007 [39]. The adherents were properly treated with common treatments in the footwear industry prior to being joined: split leather test samples were roughened at 2800 rpm with a P100 aluminium oxide abrasive cloth (Due Emme Abrasivi, Pavia, Italy) using a roughing machine (Superlema S.A., Zaragoza, Spain); SBR rubber was roughened and halogenated with 2 wt% trichloroisocyanuric acid solutions in ethyl acetate. After treating the surface of the different materials, the polyurethane adhesive was applied to each strip with a brush until the thickness of an adhesive film (132 μm) was reached and then left to air-dry for 30 min. Once the solvent had evaporated, the adhesive film remained on the substrates [40]. Joint formation was carried out after 30 min of adhesive application on 150 × 30 mm (split leather/adhesive/SBR rubber) joints. For this purpose, the adhesive was activated by infrared radiation at 80 °C in a CAN 02/01 temperature-controlled heater provided by AC&N (Elda, Spain). Thereafter, to obtain a proper bond, the strips were placed in contact with each other, and a pressure of 1.8 bar was applied for 10 s. Finally, after 72 h, the joints were formed. T-peel strength measurements were then performed in a universal testing machine Instron 34TM-10 (Instron Ltd., Buckinghamshire, UK) at a crosshead speed of 100 mm/min [41,42]. Failure modes were assessed according to ISO standard 17708 [43].

## 3. Results

**Scheme 1:**
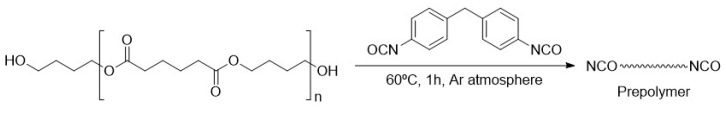
Synthesis of PU prepolymer.

**Figure 1:**
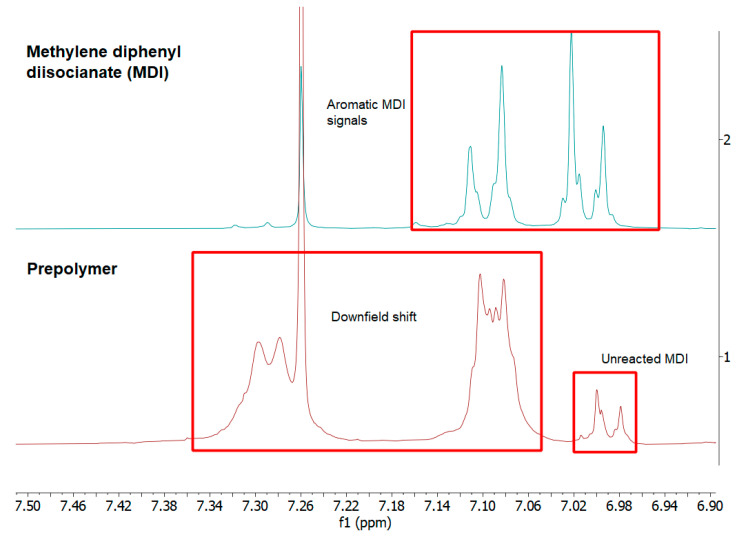
Comparison of the ^1^H-NMR spectrum (6.90–7.50 ppm, CDCl_3_, 400 MHz) of MDI and prepolymer.

Initially, the optimisation of the prepolymer synthesis was carried out by a reaction of 1,4-butanediol polyadipate with MDI under an Argon atmosphere and neat conditions, with varying temperatures and reaction times. Thus, different prepolymer batches were prepared, where the influence of temperature (between 60 and 90 °C) and time (1 to 4 h) was studied. The reaction conversions were determined by ^1^H-NMR analysis of the crude polycarbamate mixture, observing the chemical shift (Δδ) of the aromatic absorption peaks of the MDI. As shown in Figure 1, these aromatic second-order absorption peaks shifted downfield from 7.05 ppm (pure MDI) to 7.19 ppm in the prepolymer. According to this ^1^H-NMR, a conversion of 86% was obtained after 1 h, which did not improve with an increase in the reaction time. In addition, the extension of the reaction time to 3 h led to a fairly viscous prepolymer, which caused stirring problems even at 60 °C. Therefore, the conditions described in Scheme 1 were chosen as optimal for the synthesis of the prepolymer.

**Scheme 2:**
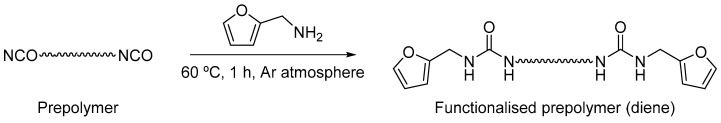
Synthesis of functionalised PU prepolymer.

**Figure 2:**
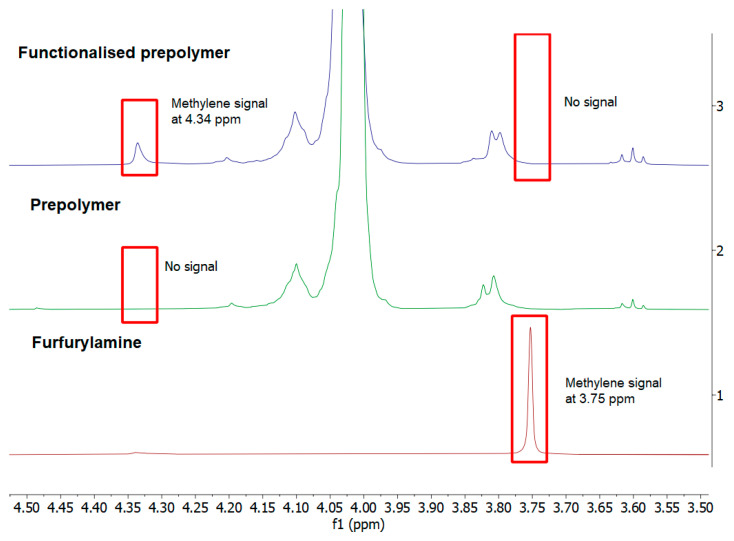
Comparison of ^1^H-NMR spectrum (400 MHz, CDCl_3_, 3.50–4.50 ppm) of furfurylamine and prepolymer before and after functionalisation.

The optimisation of the prepolymer functionalisation was carried out by adding furfurylamine to the freshly prepared prepolymer under an Argon atmosphere at different temperatures (60–90 °C) and times (1–5 h). The reaction conversion was also monitored by ^1^H-NMR, following, on this occasion, the methylene signal of furfurylamine, which suffered a downfield shift from 3.75 ppm to 4.34 when reacting with the remaining isocyanate groups, as can be observed in Figure 2 for a representative batch. All optimisation studies showed a complete conversion of furfurylamine; therefore, 60 °C and 1 h were again chosen as the optimal conditions for the prepolymer functionalisation step (Scheme 2).

**Scheme 3:**
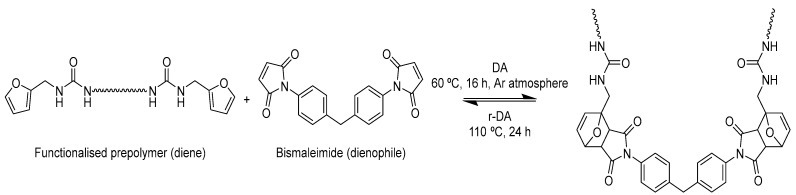
Diels–Alder and retro-Diels–Alder reactions.

The DA reaction between the functionalised prepolymer and 1,1′-(methylenedi-4,1-phenylene) bismaleimide was subsequently studied. Again, an optimisation of temperature (60–90 °C) and time (4–21 h) was carried out under net conditions, obtaining the best conversion (85%) at 80 °C after 14 h. Despite the conversion being slightly lower (82%) at 60 °C, these conditions were chosen as optimal and more sustainable for conducting the DA process (Scheme 3). Conversely, higher temperatures (90 °C) usually led to lower conversions (50%), probably because the retro-cycloaddition was starting to occur.

**Figure 3:**
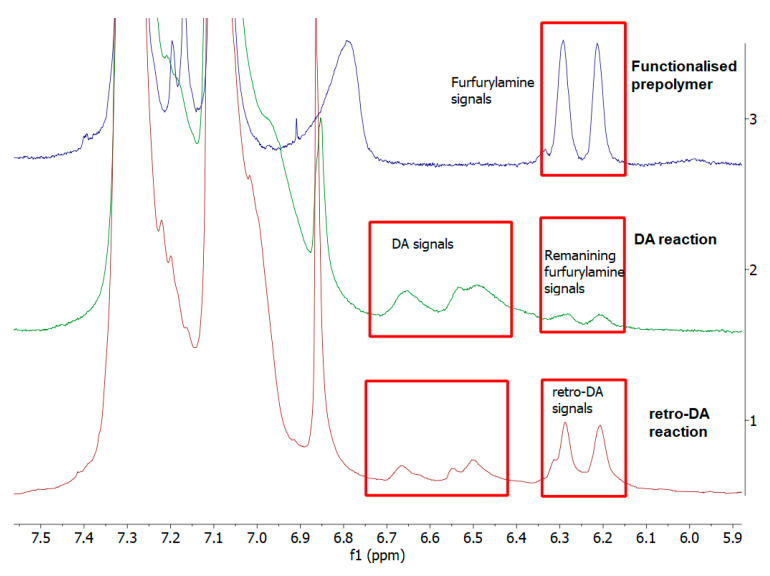
Comparison of the ^1^H-NMR spectrum of the polymer before and after DA and r-DA reactions.

The r-DA reaction was also optimised in terms of temperature and time. The optimisation was conducted at higher temperatures (90–130 °C), as required for the r-DA reactions, in the reaction vessel under conventional mechanical stirring. A good conversion to the DA adducts was observed by ^1^H-NMR at 110 °C (60% after 24 h) and 130 °C (65% after 21 h) (see Figure 3 for a representative example). Then, the optimisation of the r-DA process was also studied in film form. To this end, different samples of the DA polymer (0.5 gr) were dissolved in methyl ethyl ketone (MEK) with a polymer/MEK wt% ratio of 18/82. A small amount (2 mL) of this adhesive was placed over aluminium foil, which was heated (90–130 °C) for 24 h, since at that temperature, the solvent readily evaporates, leaving the polymer in film form over the aluminium foil. The ^1^H-NMR analysis of the different polymer batches (see Figure 3 for a representative example) was subsequently carried out by monitoring the aromatic signals of furfurylamine to determine the reaction conversion. This analysis demonstrated that 110 °C was the optimal temperature for the r-DA reaction (Scheme 3).

After optimisation of the synthesis process, four samples were characterised, and their properties were evaluated. The reference adhesive used (PU1) was the one in which, after the synthesis of the prepolymer using the optimised reaction conditions, a chain extension was conducted by incorporating 1,4-butanediol. Moreover, adhesive PU3 was prepared employing the optimised conditions. To evaluate the effect of utilising a longer reaction time and a higher temperature during the reaction with BMI, adhesive PU2 was selected. Thus, the conditions applied were 20 h at 80 °C, instead of 16 h at 60 °C. Finally, adhesive PU4 was prepared using the optimised reaction conditions, but varying the NCO/OH ratio from 1.4 to 1.1. This adhesive was therefore selected to evaluate the effect of a decrease in free NCO end groups available for functionalisation with Diels–Alder adducts. Table 1 summarises the selected adhesives as well as the conversions found for DA and r-DA reactions.

First, liquid adhesives were characterised to compare their processability. For this purpose, a determination of the solids content and viscosity was conducted, which resulted in a solids content of approximately 18 ± 2 for these adhesives. Table 2 shows the results obtained from this analysis.

Table 3 presents the Brookfield viscosity measurements of the polyurethane adhesives with and without DA adducts, performed at 25 °C. It can be observed that the incorporation of DA adducts produced a decrease in the viscosity of the polyurethane adhesives. This could be due to a decrease in the molecular weight, since DA adducts present higher steric hindrance and lower reactivity than the low-molecular-weight diol used in the reference adhesive for chain extension (1,4-butanediol). This is in line with the decrease in viscosity found in PU2, compared to PU3, for which longer reaction times and higher reaction temperatures could lead to a higher molecular weight. For PU4, the decrease in viscosity could be attributed to a lower content in free NCO end groups that are available for the functionalisation with DA adducts.

Adhesive characterisation was carried out on solid films prepared from the adhesives. For this purpose, the adhesive was placed in a Teflon mould, and the solvent was left to evaporate under standard laboratory conditions (23 °C and 50% relative humidity) for one week.

To evaluate the effect of r-DA, solid samples of each adhesive (including the reference adhesive, PU1) were kept at 110 °C for 24 h in an oven. After this time, the samples were left to cool down at room temperature and were then characterised using the different experimental techniques. In the tables and figures, all adhesives after r-DA are referred to as “PUX r-DA” to label the adhesive after being subjected to the same time–temperature schedule to activate the retro-Diels–Alder reaction.

**Figure 4:**
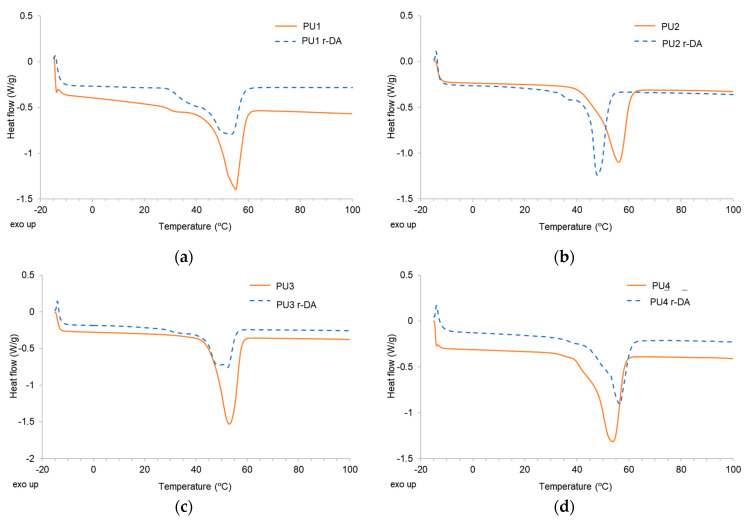
DSC thermograms (1st heating) of the adhesives before and after retro-Diels–Alder. (**a**) PU1 ref; (**b**) PU2 (conversion of 69% DA and 37% r-DA); (**c**) PU3 (conversion of 68% DA and 57% r-DA); and (**d**) PU4 (conversion of 63% DA and 62% r-DA).

The thermal behaviour of the PU adhesives before and after retro-Diels–Alder was evaluated by means of differential scanning calorimetry (DSC) and thermogravimetric analysis (TGA). A DSC analysis was conducted to compare the thermal characteristics of the reversibly connected chain segments with those of the reference polyurethane adhesive without DA adduct-based linkages. Regarding the first DSC heating run, when analysing the reference adhesive (PU1), it could be observed that the melting onset occurred at the same temperature before and after r-DA (28 °C) (Figure 4a).

In adhesives with DA adducts, PU2 and PU3 (Figure 4b and c, respectively), a decrease in the melting onset occurred after external stimulation, which caused the breaking of covalent bonds in the prepolymer in order to initiate the r-DA. This shift was less pronounced in adhesive PU2 (for which melting started at 38 °C when unactivated and at 27 °C when activated), where the reaction times and temperature of adduct incorporation were higher than in adhesive PU3 (for which melting started at 35 °C when unactivated and at 22 °C when activated).

Furthermore, when altering the NCO/OH ratio (PU4), this decrease was even less pronounced (from 33 °C to 29 °C after activation) (Figure 4d), due to the lower number of reversible covalent bond functionalisation points.

**Figure 5:**
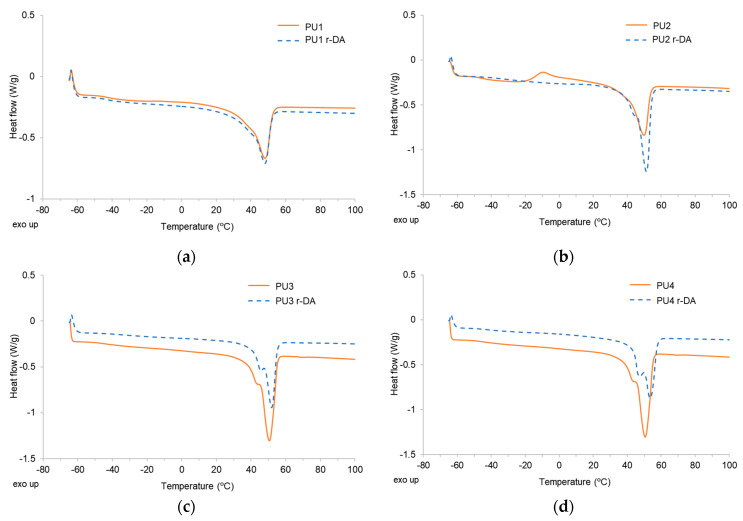
DSC thermograms (2nd heating) of the polyurethane adhesives before and after retro-Diels–Alder. (**a**) PU1 ref; (**b**) PU2 (conversion of 69% DA and 37% r-DA); (**c**) PU3 (conversion of 68% DA and 57% r-DA); and (**d**) PU4 (conversion of 63% DA and 62% r-DA).

In the second DSC heating run (Figure 5), the differences between before and after the r-DA were less noticeable, as the adhesives had been previously subjected to an initial heating. Retro-Diels–Alder occurred at around 120 °C, but it is possible that it had already begun at lower temperatures, hence the slight homogenisation after the first heating run [44,45].

A high enthalpy of fusion (in absolute value) of adhesives is often associated with a high crystallinity. In this case, the crystallinity of the functionalised adhesives decreased in the second heating run compared with the first run due to the higher cooling rate in the DSC compared to environmental conditions (23 ± 2 °C, 50 ± 5% RH), which is the temperature at which the samples were prepared (see Table 4 and Table 5). Consequently, this higher cooling rate hindered the formation of crystals, and therefore, the enthalpy associated with the melting of crystals was lower.

In addition, after comparing the effect of r-DA on the crystallinity of the functionalised adhesives, it was observed that the initial crystallinity (first heating) was lower compared to the initial crystallinity before applying r-DA conditions. This is due to the fact that this reaction resulted in the breaking of the hard segment bonds, which mainly form the crystals. However, this change was not noted in the reference adhesive, as it did not contain any breakable DA adducts. Conversely, in the second heating run, the crystallinity increased slightly again, since as previously mentioned, DSC heating can activate the formation of DA bonds.

**Figure 6:**
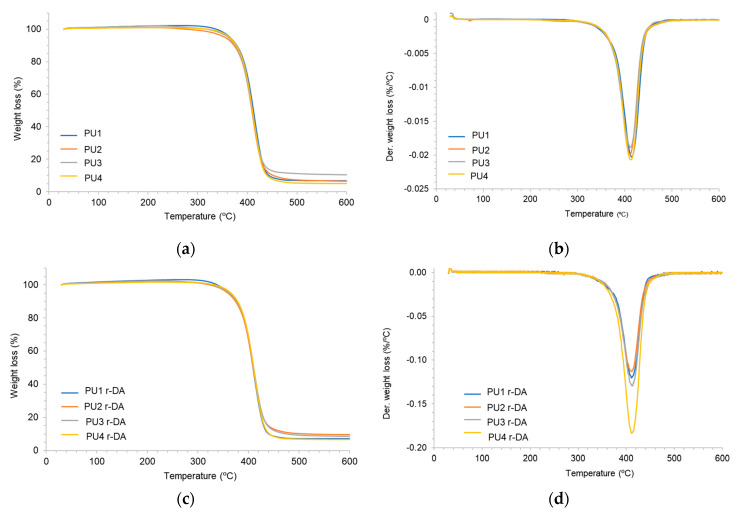
TGA and DTG curves of the polyurethane adhesives before (**a**,**b**) and after retro-Diels–Alder (**c**,**d**).

Regarding TGA, the TGA thermograms and DTG curves of polyurethane before and after r-DA are shown in Figure 6, and the results are described in Table 6. The TGA curves demonstrate that the decomposition process of polyurethanes with DA adducts in their structure was remarkably similar to the reference polyurethane adhesive without DA (Figure 6a). In all polyurethane adhesives, a single decomposition occurred at around 415 °C, which was concluded from the DTG curves (Figure 6b). The incorporation of DA adducts did not modify the thermal stability of polyurethane adhesives. After performing the r-DA on polyurethane adhesives, the thermal stability remained constant (Figure 6c,d). There were no major changes in decomposition temperatures and weight loss, as depicted in Table 6. This performance of polyurethanes with DA adducts is also endorsed by the literature [12,46,47].

In the footwear industry, the most demanding joint is the upper-to-sole joint, since it is subject to high stress and may be exposed to environmental conditions that can alter it. Therefore, high adhesive forces are required to withstand it.

A method to strengthen this bond is to incorporate a crosslinking additive, thereby rendering the bond thermosetting. Consequently, T-peel strength tests were carried out using leather/adhesive with and without crosslinker/SBR rubber joints, which serve as a representative model for the upper-to-sole joint in footwear. Samples with a crosslinker were prepared by adding 5 wt% of a commercial isocyanate, consisting of p-isocyanate-phenyl tri-thiophosphate, to the adhesive immediately before application to the substrates. Adhesion was evaluated after 5 min of joining both substrates (initial adhesion), after 72 h (final adhesion) and after heating the joints at 110 °C for 24 h to activate the r-DA reaction. In the latter case, the joints were removed from the oven and left to cool down to room temperature prior to performing the T-peel test.

**Figure 7:**
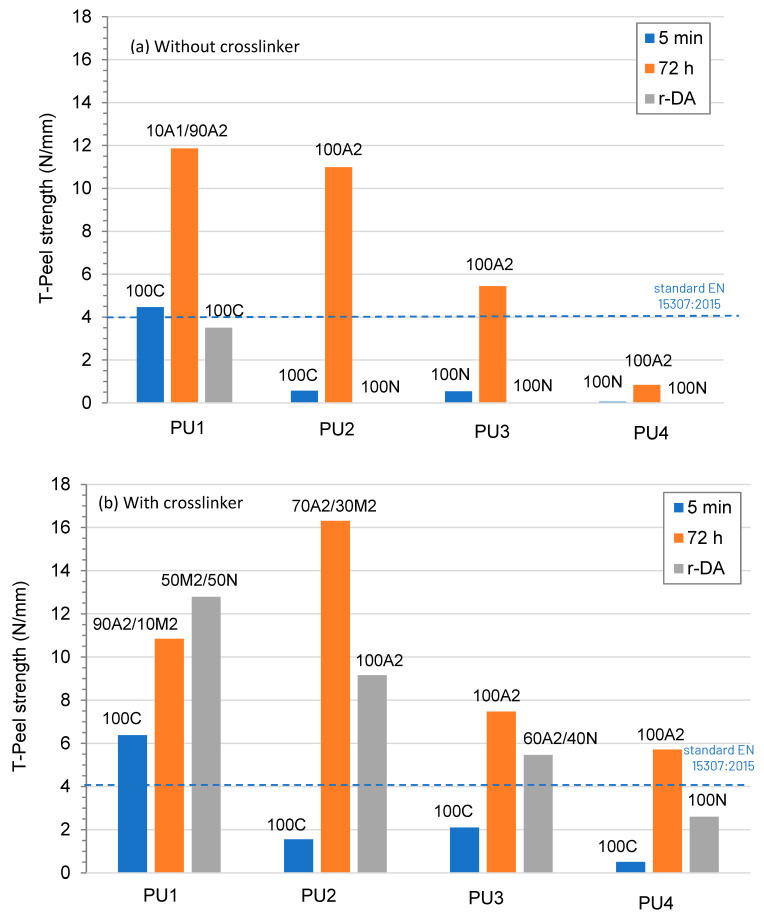
(**a**) T-peel strength values of leather/PU adhesives with DA adducts/SBR joints after 72 h and after r-DA without crosslinker. (**b**) T-peel strength values of leather/PU adhesives with DA adducts/SBR joints after 72 h and after r-DA with crosslinker. Failure results are given in percentages, where there may be a mixture of failures (e.g., 10A1/90A2) or where only one type of failure occurred (100C). Types of failure: M2: tear of the rubber; A2: the adhesive is peeled from the rubber; N: cohesive failure in the adhesive, without threads; and C: cohesive failure in the adhesive, with threads.

A loss of initial adhesion was observed when functionalising the adhesive with DA adducts, which was particularly pronounced when no crosslinker was utilised (Figure 7a,b) [47]. For adhesives PU2 and PU3, with and without a crosslinker, a good final adhesion was obtained, meeting the minimum strength requirements set by EN 15307:2015 for upper-to-sole bonding: a minimum of 5 N/mm (3.5 N/mm with substrate failure) for high-demand footwear (mountain) or 4 N/mm (3 N/mm with substrate failure) for medium-demand footwear (children’s and general purpose sports) [48]. However, adhesive PU4 did not meet these requirements unless used with a crosslinker. The initial failures observed in the joint with and without a crosslinker were cohesion failures. However, when the final adhesion was accomplished, the failure changed to the adhesive being peeled from the rubber as a consequence of tearing, whether they contained a crosslinker or not.

**Figure 8:**
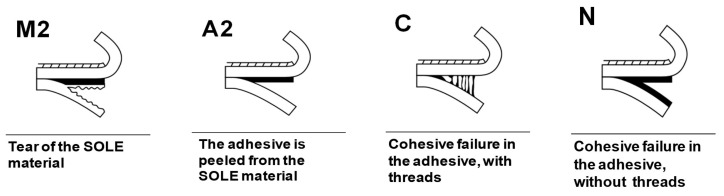
Representative types of failure after separation of adhesive joints..

Once the r-DA reaction was activated in the adhesive bonds, a sharp drop in adhesion was observed in functionalised adhesives PU2, PU3 and PU4 when no crosslinker was employed. This was not the case for the reference adhesive (PU1), which still presented considerable adhesion and did not allow disassembly. As expected, the incorporation of a crosslinker in the adhesive increased the bond strength, which remained high in adhesives PU2 and PU3 after activating the r-DA reaction and breaking the covalent bonds. However, it should be noted that, in these formulations, adhesion decreased after activation, in contrast to the reference adhesive, where a slight increase was observed. In relation to adhesive PU4, which already started from a moderate adhesion even in the presence of a crosslinker, a drop in adhesive strength was also noticed after activating the r-DA reaction, but not as significant as in the formulation without a crosslinker. Against these results, it can be concluded that, after r-DA reaction, there is a general tendency to obtain cohesive failures in the functionalised adhesives. Figure 8 shows the representative types of failures that occurred after the debonding of the adhesive joints.

## 4. Conclusions

A series of new polyurethane adhesives, functionalised with furfurylamine and BMI, were synthesised, confirming that reversible bonds based on a Diels–Alder reaction can be introduced in polyurethane networks. Different reaction times, reaction temperatures and NCO/OH ratios were studied. A 100% conversion (followed by H^1^-NMR) was obtained in the functionalisation of the polyurethane prepolymer with furfurylamine. For the Diels–Alder reaction with BMI, the conversion was 63–69%, depending on the reaction time, temperature and NCO/OH ratio. Then, by optimising the synthesis and activation conditions, a conversion of up to 57% was obtained for the retro-Diels–Alder reaction, which represents a major milestone in the improvement of this process. Moreover, the importance of these reactions lies in the fact that they occurred without the addition of solvents, and therefore contribute to the sustainability of the production process.

The functionalisation of the PU adhesives with Diels–Alder adducts led to an increase in the viscosity of the prepolymer during the synthesis, which hampered the incorporation of a chain extender, as is common for non-functionalised adhesives. This produced a decrease in the final viscosity of the adhesive compared to the that of the reference adhesive prepared with the same solids content, which may be due to a lower molecular weight.

The thermal properties of PU adhesives were analysed by differential scanning calorimetry (DSC) and thermogravimetric analysis (TGA). The incorporation of DA adducts in the prepolymer of polyurethane did not change the thermal resistance of the adhesives. However, DSC experiments showed a decrease in the melting onset of the functionalised adhesives after the activation of r-DA, which demonstrates that there was a certain degree of breaking of covalent bonds.

A loss of initial adhesion was observed when functionalising the adhesive with DA adducts, which was particularly pronounced when no crosslinker was used. Functionalisation did not negatively affect the final adhesion of the polyurethane adhesives, which remained within the requirements established by the standard for upper-to-sole adhesive joints in footwear. The breakage of bonds produced during the r-DA reaction led to a decrease in the adhesive strength of the bonds with functionalised adhesive, preceding cohesion failures. This would allow for the disassembly of the joint, thus enabling the separation and recycling of the substrates. However, when the adhesive was used together with a crosslinker, the effect of r-DA was less prominent. Therefore, a subsequent optimisation of the adhesive synthesis process was then required.

These findings mark the beginning of future research in polyurethane adhesive functionalisation, focusing on enhancing and streamlining the Diels–Alder and retro-Diels–Alder processes by refining reaction parameters, reducing time, and optimising temperatures. In terms of sustainability, this research paves the way for progress in adhesive design towards greener and more adaptable adhesive materials for diverse applications.

## Figures and Tables

**Table 1:** Nomenclature and conversion percentages of the adhesives under study.

Adhesive	NCO/OH	DA Conversion (%)	r-DA Conversion (%)
PU1	1.4	-	-
PU2	1.4	69%	37%
PU3	1.4	68%	57%
PU4	1.1	63%	62%

**Table 2:** Solids content (UNE-EN 827).

Adhesive	Solids Content (wt%)
PU1	16.6 ± 0.1
PU2	16.3 ± 0.1
PU3	17.7 ± 0.04
PU4	17.6 ± 0.1

**Table 3:** Brookfield viscosity (25 °C) (cPs).

Speed (rpm)	PU1	PU2	PU3	PU4
10	250	10	100	30
20	245	15	100	30
30	247	20	103	30
50	246	26	108	36
60	248	28	110	40
100	249	36	120	49

**Table 4:** Results from DSC (1st heating run) of the adhesives before and after the r-DA.

Adhesive	T_m_ (°C)	ΔH_m_ (J/g)	Adhesive	T_m_ (°C)	ΔH_m_ (J/g)
PU1	55	−54.48	PU1 r-DA	53	−33.97
PU2	56	−47.70	PU2 r-DA	48	−38.27
PU3	53	−57.84	PU3 r-DA	52	−36.13
PU4	54	−55.60	PU4 r-DA	56	−39.13

**Table 5:** Results from DSC (2nd heating run) of the adhesives before and after the r-DA.

Adhesive	T_m_ (°C)	ΔH_m_ (J/g)	Adhesive	T_m_ (°C)	ΔH_m_ (J/g)
PU1	48	−33.51	PU1 r-DA	48	−32.54
PU2	50	−34.65	PU2 r-DA	51	−45.57
PU3	50	−43.50	PU3 r-DA	52	−35.40
PU4	50	−52.50	PU4 r-DA	53	−43.48

**Table 6:** Comparison of TGA and DTGA results before and after r-DA.

Adhesive	T_d_ (֯C)	Δm_d_ (%)	Residue (%)	Adhesive	T_d_ (֯C)	Δm_d_ (%)
PU1	415	95	5	PU1 r-DA	410	95
PU2	411	94	6	PU2 r-DA	409	93
PU3	412	90	10	PU3 r-DA	412	94
PU4	413	95	5	PU4 r-DA	411	95

## Data Availability

The data presented in this study are available upon request from the corresponding author.
